# A review of the multifunctionality of angiopoietin-like 4 in eye disease

**DOI:** 10.1042/BSR20180557

**Published:** 2018-09-14

**Authors:** Xinyue Yang, Yan Cheng, Guanfang Su

**Affiliations:** Department of Ophthalmology, The Second Hospital of Jilin University, Changchun 130041, China

**Keywords:** ANGPTL4, angiogenesis, inflammation, ocular diseases, vascular permeability

## Abstract

Angiopoietin-like protein 4 (ANGPTL4) is a multifunctional cytokine regulating vascular permeability, angiogenesis, and inflammation. Dysregulations in these responses contribute to the pathogenesis of ischemic retinopathies such as diabetic retinopathy (DR), age-related macular degeneration (AMD), retinal vein occlusion, and sickle cell retinopathy (SCR). However, the role of ANGPTL4 in these diseases remains controversial. Here, we summarize the functional mechanisms of ANGPTL4 in several diseases. We highlight original studies that provide detailed data about the mechanisms of action for ANGPTL4, its applications as a diagnostic or prognostic biomarker, and its use as a potential therapeutic target. Taken together, the discussions in this review will help us gain a better understanding of the molecular mechanisms by which ANGPTL4 functions in eye disease and will provide directions for future research.

## Introduction

Angiopoietin-like protein 4 (ANGPTL4) is a secreted protein and a member of a family of angiopoietin-like proteins (ANGPTL1–8). It was initially discovered in 2000 by three independent research groups as a regulator of lipid metabolism-induced by peroxisome proliferator activated receptor (PPAR) γ (PPARγ) under fasting conditions, regulating namely fasting-induced adipose factor (FIAF), hepatic fibrinogen/angiopoietin related protein (HFARP), and peroxisome proliferator-receptor-γ angiopoietin-related (PGAR) [1–3]. Recent studies have shown that ANGPTL4 regulates tumorigenesis, angiogenesis, vascular permeability, glucose homeostasis, lipid metabolism, cell differentiation, energy homeostasis, wound healing, inflammation, and redox regulation [4]. The purpose of this review is to describe the regulatory effects of ANGPTL4-associated pathways and provide information for the potential development of this protein as a clinical treatment target in eye disease therapy.

## ANGPTL4: structure and expression patterns

ANGPTL4 is a part of a superfamily of angiopoietins that share a similar structure and carry out related functions that contain N-terminal coiled-coil domain (CCD) connected to a C-terminal fibrinogen-like domain (FLD) via a cleavable linker [5]. ANGPTL4 shares high sequence homology with ANGPTL3 and ANGPTL8 [6]. The human *ANGPTL4* gene is well preserved amongst various species [7]. The human *ANGPTL4* gene is located on chromosome 19p13.3 and encodes a 45–65 kDa glycoprotein. The molecular mass discrepancy reported amongst several studies is probably due to the different glycosylation, oligomerization, and cleavage forms which may be a result of the specific cell line/type used in the study. This protein has some N- and O-glycosylation sites and was confirmed to be N-glycosylated at amino acid position 177 [8]. Ruddock and co-workers [9] provided the first evidence of the crystal structures of the FLDs of ANGPTL4 protein. The FLDs of ANGPTL4 form compact structures and is split into three subdomains A, B, and P. The N-terminal domain (subdomain A) is the most conserved amongst the FLD containing homologs and superimposes well in ANGPTL4, subdomain B is the largest subdomain amongst the three subdomains, the subdomain P varies the most amongst FLD containing proteins which functions as a site for ligand binding. There are obviously differences in the structures of subdomain P between ANGPTL3 and ANGPTL4 indicating that while loss-of-function mutations, ANGPTL3 and ANGPTL4 act by different pathways [9].

Previously, ANGPTL4 was considered to be an orphan ligand because it does not bind to the angiopoietin receptor tyrosine kinase Tie2 and Tie1; information about its likely binding partner was lacking [10]; while till now emerging evidence has indicated that ANGPTL4 has many binding partners, such as integrin β1, integrin β5 [11] and integrin αvβ3 [12], lipoprotein lipase (LPL) [13], syndecans [14], cadherin-5 [15] and cadherin-11 [16]. ANGPTL4 assembles into dimers and tetramers in cells, and two cysteine residues (Cys^76^ and Cys^80^) in the N-terminal domain are crucial to the stability of intermolecular disulphide bonds in ANGPTL4 oligomers [17]. The full-length ANGPTL4 protein is proteolytically processed in the linker region (a major cleavage site between Lys^168^ and Lev169, a minor cleavage site between Lys^170^ and Met^171^ [18]) by proprotein convertases. The cleavage of ANGPTL4 is tissue dependent. Various tissues produce ANGPTL4 which is secreted into the bloodstream in glycosylated, oligomerized, native, and cleaved isoforms. ANGPTL4 is expressed in the plasma at 60 kDa in various forms. Adipose tissue secretes full-length ANGPTL4, while the liver secretes nANGPTL4 isoforms [19]. Immunoblot analyses demonstrated that ARPE-19 cells secrete an approximately 55-kDa full-length ANGPTL4 [20]. ANGPTL4 contributes to proteolytic processing and oligomerization. Different studies have shown that the nANGPTL4 domain is used to modulate lipid metabolism, whereas the cANGPTL4 domain may be a modulator of tumorigenesis [21].

Recent research has demonstrated that physiological conditions, such as fasting conditions, hypoxia, pregnancy and lactation, and adipocyte differentiation result in ANGPTL4 up-regulation [22–24]. Furthermore, chronic caloric restriction, short-term cooling, very low-calorie diet (VLCD), high-fat, high-energy diet (HFED), and free fatty acids (also called NEFA) have been shown to increase plasma concentrations of ANGPTL4 [25–27]. Transcription factors such as forkhead box O1 (FOXO1), hypoxia inducible factor-1 α (HIF-1α), PPARs, single-minded homolog 1 (SIM1), aryl-hydrocarbon receptor nuclear translocator (ARNT), pleiomorphic adenoma gene-like 1 (PLAG1), endothelial PAS domain protein 1 (EPAS1), nuclear factor‐κB (NF‐κB), muscle RING‐ finger protein‐1 (MURF1), c-Myc and glucocorticoid receptor gene (NR3C1) could enhance ANGPTL4 expression through different mechanisms [28–36].

Using microarray and ChIP-seq analysis, Inoue et al. [37] first reported that two transcription factors, HIF1 and PPAR β/δ, utilize synergistic transcriptional regulation via a conformational change to their common target gene *ANGPTL4*. Using genome-wide transcriptional profiling technology, Kaddatz et al. [38] reported that human ANGPTL4 expression might be synergistically induced by the functional interactions of transforming growth factor-β (TGF-β) and PPARβ/δ signaling. Agonists such as PPAR agonists, protein kinase C (PKC) agonists, retinoic acid receptors (RXR) agonists, HIF-1α agonists, glucocorticoid receptor agonists, and angiotensin receptor blockers, drugs such as paeoniflorin, glucocorticoids, chiglitazar, fibrates, thiazolidinediones, PMA, 1,2-dioctanoyl-sn-glycerol (DOG), bryostatin, L-mimosine (L-MIM), advanced glycation end products (AGEs) and dexamethasone [32,39–43], inhibitors of known receptors (such as angiotensin blockers, resistin, leptin, and insulin [44]), and inflammation molecules (such as lipase, tumor necrosis factor-α, interleukin-1β, and interferon-γ [45,46]) were shown to regulate the expression of ANGPTL4. HIF-1α induction of ANGPTL4 under hypoxic conditions was first described in cardiomyocytes but also occurs in other cell types including adipocytes, endothelial cells, chondrocytes, monocytes, musculoskeletal cells, osteoclasts, and osteoblasts [47]. The promoter region of ANGPTL4 had high levels of promoter methylation, demethylating agents and a histone deacetylase inhibitor that could restore ANGPTL4 expression. Recently, genome-wide analyses of the mammalian transcriptome have revealed an important class of transcripts – non-coding RNAs. Sun and co-workers [48] demonstrated that the long non-coding RNA PVT1 inhibits ANGPTL4 transcription through binding with the enhancer of zeste homolog 2 (EZH2) in trophoblast cell.

ANGPTL4 is abundant in adipose tissue and the vascular system, but also in other organs, such as the liver, intestine, brain, thyroid, eye, kidney, heart, skeletal muscles, spleen, pituitary gland, hypothalamus, and placenta [2,6,22,49–55].

## Functions of ANGPTL4

**Figure 1:**
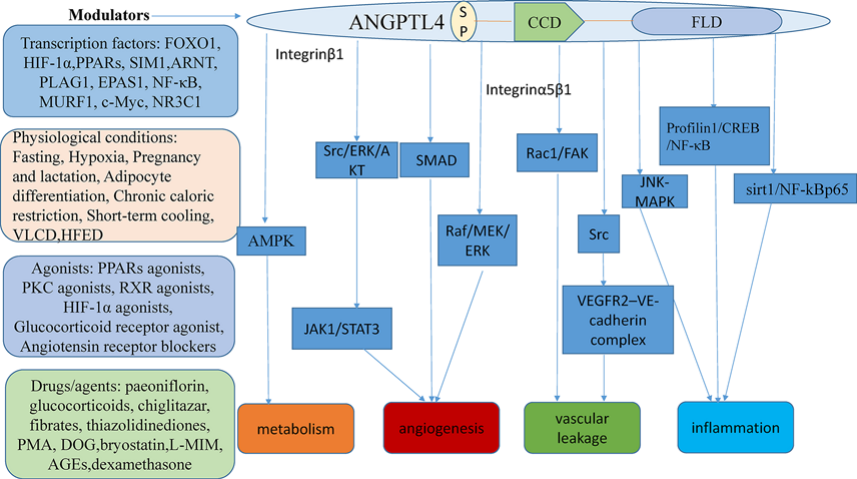
ANGPTL4 function associated with the pathogenesis of metabolism, angiogenesis, and inflammation Abbreviation: SP, signal peptide.

ANGPTL4 is a multifunctional secreted protein, involved in many physiological processes with a variety of effects upon human health and disease. Here, we review the aberrant expression of ANGPTL4 and summarize the specific pathogenic functions and possible mechanism by which these functions occur (Figure 1).

### Regulation of lipid and glucose metabolism

ANGPTL4 regulates circulating triglyceride-rich lipoproteins, very low-density lipoproteins (VLDL), and chylomicrons by irreversibly inhibiting LPL and ANGPTL4 is present in high-density lipoprotein (HDL). Studies have revealed that ANGPTL4 is induced early in fasting to transfer fatty acids and triglycerides from adipose tissue [56]. In type 2 diabetes, circulating ANGPTL4 is increased to protect HDLs from hydrolysis [57]. ANGPTL4 inhibits LPL activity and FLD of ANGPTL4 stimulates the lipolysis of triacylglycerol stored by adipocytes in white adipose tissue (WAT) [58]. In addition, the intestinal microbiota represses ANGPTL4 expression in fat storage [50]. Recent research also demonstrated that ANGPTL4 promotes bile acid (BA) absorption via the gut microbiota mechanism [59]. Later, Larsen and co-workers [60] pointed out that ANGPTL4 can not be effectively regulated by modifying the gut microbiota composition. Furthermore, E40K nucleotide polymorphisms of the *ANGPTL4* gene decreased oligomer formation, which is correlated with decreased LPL inhibition activity and significantly lower triglyceride levels [17].

ANGPTL4 also participates in the flux of lipids from the WAT to the liver [61]. A positive correlation between increased ANGPTL4 and non-esterified fatty acids levels in the plasma of healthy subjects after different dietary regimens has been shown [26]. Previous study demonstrated that patients with type 2 diabetes have lower plasma ANGPTL4 concentrations than the healthy group. Studies of transgenic mice showed that ANGPTL4 decreases blood glucose and improves glucose tolerance, at the same time, it induces hypertriglyceridemia and hepatic steatosis [19]. General contributions of ANGPTL4 to dyslipidemia and coronary artery disease (CAD) has also been demonstrated [62–65].

Muendlein et al. [65] demonstrated that plasma ANGPTL4 was higher in patients with metabolic syndrome, and the number of single nucleotide polymorphisms in ANGPTL4 could predict future cardiovascular events. Further study showed that carriers of inactivating genetic variants of ANGPTL4 had lower triglyceride [66] levels and CAD risk, suggesting that ANGPTL4 might be a possible therapeutic target for the treatment of ischemic heart disease [67]. Jabs et al. [68] demonstrated that endothelial Notch signaling modulates ANGPTL4 expression which damages fatty acid transport and leads to vascular remodeling of the adult heart. A recent study showed that ANGPTL4 participated in hypothalamus regulation of energy metabolism attributed to the suppression of adenosine 5′-monophosphate (AMP)- activated protein kinase (AMPK) activities [55]. Clement et al. [69] demonstrated that through negative feedback loops, ANGPTL4 is linked to proteinuria and hypertriglyceridemia in nephrotic syndrome and ANGPTL4 also plays an important role in the barrier function of glomerular basement membrane (GBM).

### Angiogenesis, tumorigenesis, and vascular permeability

Current studies demonstrate that the role of ANGPTL4 in angiogenesis is controversial as angiogenic effects and anti-angiogenic effects have both been reported [70,71]. Recently, mounting evidence has suggested that ANGPTL4 seems to be a proangiogenic factor. Hermann et al. [72] reported that ANGPTL4 was highly expressed in the early stage of collagen-induced arthritis and recombinant mouse ANGPTL4 played a role in angiogenesis in human umbilical vein endothelial cells (HUVECs). Chong et al. [70] demonstrated that recombinant cANGPTL4 accelerated wound re-epithelialization in diabetic mice by stimulating wound angiogenesis. They reported that ANGPTL4 regulated angiogenesis via keratinocyte-to-endothelial cell communication. ANGPTL4-induced nitric oxide production through integrin/JAK/STAT3-mediated up-regulation of inducible nitric oxide synthase expression in wound epithelia [70]. Sodhi and co-workers** [73]** demonstrated that recombinant human ANGPTL4 stimulates immortalized human dermal microvascular endothelial cells (HMEC-1) tubule formation *in vitro* in a dose-dependent manner and stimulates corneal neovascularization *in vivo*. On the other hand, ANGPTL4 seems to be an anti-angiogenic factor. Cazes et al. [71] provided the first evidence that ANGPTL4 reduces HUVECs migration and decreases tube formation and sprouting of HMEC-1 cells. Ushijima and co-workers [74] demonstrated that ANGPTL4 inhibits vascular tube formation and proliferation of HUVECs due to inhibition of the extacellular signal-regulated kinase (ERK) signaling pathway. Yang et al. [18] demonstrated that the C-terminal end of ANGPTL4 alone is essential for the inhibition of angiogenesis through the Raf/MEK/ERK1/2 MAPkinase pathway in HUVECs. Furthermore, N-glycosylation of C-ANGPTL4 contributes to its anti-angiogenic activities. Chomel et al. [75] demonstrated that the N-terminal domain of ANGPTL4 binds to the extracellular matrix (ECM) in endothelial cells and plays a role in the inhibition of hypoxic HUVECs adhesion, migration, and sprouting. Furthermore, the glycosylated C-terminal FLD of ANGPTL4 was shown to play anti-angiogenic role, both the N-terminal and C-terminal of ANGPTL4 did not display anti-angiogenic properties [75]. Until now, we cannot explain the discrepancy between these evidence, it is highly possible that the function of ANGPTL4 in modulating angiogenesis differs in different tissue contexts and also involves in its binding partner, release, and proteolysis. There is a large amount of research showing that various factors induce ANGPTL4 function in angiogenesis. Padua et al. [76] demonstrated that TGF-β induces ANGPTL4 expression via the Smad signaling pathway which plays a role in tumor metastasis. Furthermore, other factors such as placental growth factor (PlGF), hepatocyte growth factor (HGF), vascular endothelial growth factor (VEGF), and platelet-derived growth factor (PDGF) were shown to induce ANGPTL4 function in angiogenesis [77–79].

Numerous published studies have signified that ANGPTL4 has an essential role in cancer onset, progression, metastasis, and anoikis resistance. High levels of ANGPTL4 are associated with a poor prognosis in solid tumors, such as prostate cancer, melanoma, hepatocellular carcinoma, bladder cancer, scirrhous gastric cancer, giant cell tumor, oral tongue cancer, and tongue squamous cell carcinoma [80–88]. Tan et al. [89] found that T266M cANGPTL4 bound to integrin α5β1 contributed to the weaker activation of downstream signaling molecules, leading to reduced proliferation, anoikis resistance, migratory capability, and impaired adenylate energy charge. In urothelial carcinoma, ANGPTL4 exerted both oncogenic and tumor-suppressive roles, furthermore, circulating ANGPTL4 level was a biomarker of tumor progression. In breast cancer, knockdown of ANGPTL4 had no effect on tumor metastasis in local lymph nodes and bone, but could inhibit metastasis in the lung [76]. ANGPTL4 may be used to indicate prognosis in hepatocellular carcinoma patients. Li et al. [90] demonstrated that ANGPTL4 promotes transendothelial migration and metastasis of hepatocellular carcinoma by stimulating vascular cell adhesion molecule-1 (VCAM-1) and activation of the VCAM-1/integrin β1 axis. Specifically, hypoxia can induce expression of the prostaglandin E2 (PGE2) receptor EP1, in colorectal cancer. PGE2 binds to EP1 and activates the EP1 signaling pathway, which in turn promotes tumor expression of ANGPTL4 and cANGPTL4 via activation on transcriptional activator (STAT) 1 (STAT1). This signaling pathway in turn promotes tumor growth and proliferation in colorectal cancer. The present study further showed that cANGPTL4 mainly regulates tumor cell proliferation, and induces STAT1 production depending on sarcoma gene (Src) /MAPK signaling pathway activation [91]. These conclusions are consistent with the findings of Zhu et al. [92] who suggested that secreted cANGPTL4 binds to integrin β1/β5 and activates focal adhesion kinase (FAK) and ras-related C3 botulinum toxin substrate (Rac1), and then activates Src through PI3K/PKRα and ERK signaling, ultimately promoting tumor growth. Germain and co-workers [93] reported that use of *in vivo* DNA electrotransfer overexpressing ANGPTL4, 3LL cells expressed less lung metastasize, suggesting that ANGPTL4 exert a role in preventing metastasis. Kirsch et al. [94] observed that ANGPTL4 binds to syndecans to forms a ternary complex with Wnt co-receptor lipoprotein receptor-related protein 6 (LRP6) which has a role as a Wnt signaling antagonist. Yao and co-workers [95] demonstrated that ANGPTL4 up-regulates bone morphogenetic protein 7 (BMP7), therefore, inhibiting apoptosis of colorectal cancer cells and promoting metastasis. Montaner and co-workers [96] demonstrated that in Kaposi’s sarcoma, viral G-protein-coupled receptor (vGPCR) might promote angiogenesis and vascular permeability by up-regulation of ANGPTL4. Therefore, the expression and mechanism of ANGPTL4 may be related to tumor type. Tarhoni et al. [97] first developed custom immunobead assays for several mechanistically important targets and evaluated these against sera from a patient cohort with non-small cell lung cancer (NSCLC). They successfully developed and analytically validated a triplex immunobead assay for the quantitation of midkine, syndecan-1, and ANGPTL4 from patient sera which will be an important tool for future studies delineating the role of angiogenesis in lung cancer progression [97].

The endothelial barrier sustains vascular and tissue homeostasis and contributes to many physiological processes. Disruption in barrier function can stimulate tissue damage during disease progression. Although the molecular mechanisms related to vascular leakage have been studied for years, recent evidence has identified new therapeutic targets that have begun to show preclinical promise. Despite plenty of evidence that has implicated the role of ANGPTL4 in cancer metastasis, the role of ANGPTL4 in vascular integrity remains unclear. Few studies have indicated that abnormalities in ANGPTL4 expression are extending to play key roles in modulating vascular integrity. However, its precise role in vascular biology remains debatable. Padua et al. [76] demonstrated that human recombinant ANGPTL4 and cells overexpressing ANGPTL4 cause acute disruption of HUVEC endothelial cell–cell junctions. Huang et al. [15] reported that recombinant cANGPTL4 associated with the adhesive domains of VE-cadherin and claudin-5, contributing to their declustering and internalization, translocation of β-catenin to the nucleus, and a leaky endothelium. Besides cANGPTL4 induces endothelial disruption by binding and activating integrin α5β1-mediated Rac1/PAK signaling [15]. While, Germain and co-workers [98] proposed ANGPTL4 as a therapeutic approach aimed at decreasing cerebrovascular permeability during stroke. They demonstrated that in a mouse model of ischemic stroke, recombinant human ANGPTL4 decreased VEGFR2–VE-cadherin complex disruption via Src signaling, which increased endothelial cell barrier integrity in the cerebral microcirculation [98].

### Inflammation

Compelling evidence has indicated that ANGPTL4 is involved in many inflammation-associated diseases. ANGPTL4 was induced by interleukin (IL)-1β (IL-1β) treatment via the JNK-MAPK signaling pathway in murine MC3T3-E1 osteoblastic cells [99]. Increased expression of ANGPTL4 in osteoarthritis has been reported, furthermore, matrix metallopeptidase (MMP) expression is induced by ANGPTL4 and played a role in cartilage matrix remodeling. These results suggest that in osteoarthritis ANGPTL4 is a potential factor in pathogenic cartilage destruction [100,101]. Guo et al. [102] demonstrated that ANGPTL4 siRNA promoted sirtuin 1 (SIRT1) expression and inhibited the nuclear factor κB (NF-κB) p65 pathway, suggesting that *ANGPTL4* is an essential gene for the treatment of lipopolysaccharide (LPS)-induced acute lung injury [102]. Schumacher et al. [103] identified ANGPTL4 as an up-regulated protein during inflammatory conditions in the bone marrow of mice and determined recombinant ANGPTL4 plays a vital role during early and late stages of hematopoieticon, specifically megakaryopoietic, reconstitution following stem cell transplantation [103]. Higher ANGPTL4 levels were reported after exposure to interleukins, tumor necrosis factor-α, interferon-γ, and prostaglandins in 3T3-L1 adipocytes [45,46]. Lichtenstein et al. [104] reported that in peritoneal macrophages incubated with chyle, ANGPTL4 reduced macrophage foam cell formation, inflammatory factors expression, and chyle-induced activation of the endoplasmic reticulum (ER) stress pathway, contributing to protection against the pro- inflammatory effects of dietary saturated fat. This indicates that ANGPTL4 is a crucial regulator of macrophage functions. Later, Aryal et al. [105] addressed the direct role of macrophage ANGPTL4 during atherogenesis. They demonstrated that ANGPTL4 reduces the progression of atherosclerosis by regulating the net blood monocyte content in lipid-rich conditions, suppressing lipid overloading in macrophages and preventing generation of foam cells and inflammation [105]. Phua et al. [106] showed that ANGPTL4 regulated the expression of tristetraprolin (TTP), an mRNA destabilizing agent, via the activation of cAMP-response element binding protein (CREB) and NF-κB in human colonic epithelial cells, indicating that ANGPTL4 may be targetted to influence cell infiltration via TTP-mediated chemokine mRNA stability [106]. These results suggest that ANGPTL4 may exert both anti- and pro-inflammatory effects, the mechanisms by which ANGPTL4 modulates inflammation requires further exploration.

## ANGPTL4 as a biomarker in various ocular diseases

The target of current treatments for neovascular retinopathy is direct inhibition of VEGF. Although most treatments achieve sufficient results, there are many patients who are insensitive to anti-VEGF therapy. The ensuing problems associated with anti-VEGF therapy also deserve attention because VEGF produced by the retinal pigment epithelium is essential to maintaining the health and homeostasis of the choriocapillaris, vascular bed, and photoreceptor cell layer [107]. This growth factor also exerts a role as a neurotrophic factor in the neurosensory retina [108]. Several compounds that target VEGF have been tested and demonstrate unparalleled effects in randomized clinical trials preventing vision loss, retinal atrophy, and glaucoma in the majority of patients with neovascularization [63,109,110]. There are other factors that contribute to neovascular (NV) formation, growth, and persistence, including members of the PDGF family, epidermal growth factor family, angiopoietin-like family, and so on [111]. Here, we review the aberrant expression of ANGPTL4 in eye diseases and summarize specific its pathogenic functions and possible mechanisms (Table 1).

**Table 1:** ANGPTL4 dysregulation involved in various eye diseases

Disease	Dysregulation	Tissue and cell type	Pathogenic functions	References
Diabetic eye disease	Up-regulated	MIO-M1 cells/Müller cells, HMEC-1 cells, OIR mice, aqueous and vitreous from DM patients, retinas of DM rats	Promotes EC proliferation, migration, and tube formation *in vitro*	[52,115–117]
		ARPE-19, human retinal endothelial cells	Increases retinal microvascular permeability *in vivo*	[20]
Branch retinal vein occlusion	Up-regulated	Aqueous from BRVO patients	Biomarker for the severity of retinal ischemia and vascular hyperpermeability	[120]
Branch retinal artery occlusion	Up-regulated	Vitreous from BRAO patients	Increases retinal neovascularization *in vivo*	[121]
Age-related macular degeneration	Up-regulated	Plasma and aqueous from AMD patients	Potential diagnostic and therapeutic biomarker in AMD	[123,124]
Sickle cell retinopathy	Up-regulated	Retina, aqueous and vitreous of PSR patients	Contribute to the development of pathological angiogenesis in PSR	[126]
Apterygium	Up-regulated	Surgically excised pterygia	Contribute to the angiogenic phenotype of pterygia	[128]
Uveal melanoma	Up-regulated	UM cell lines, MCTS	Participate in the promotion of angiogenesis in UM	[130,131]

Abbreviations: AMD, age-related macular degeneration; BRAO, branch retinal artery occlusion; BRVO, branch retinal vein occlusion; DM, diabetes mellitus; MCTS, multicellular tumor spheroid; PSR, proliferative sickle cell retinopathy; UM, uveal melanoma.

### Diabetic retinopathy

Diabetes mellitus (DM), a condition where a patient has fasting plasma glucose values ≥ 7.0 mmol/l (126 mg/dl), is increasingly common throughout the world. DM is now recognized as a common pathophysiology involving metabolic disorders, oxidative impairment, and vicious cycles that aggravate organ dysfunction that drives the diabetic state throughout its disease course and clinical presentations. Diabetes poses an increased risk for cardiovascular disease, kidney failure, and blindness [112]. Diabetic retinopathy (DR) is a common cause of vision impairment, affecting 93 million people globally. Vision loss in DR prominently contributes to diabetic macular edema (DME) but may also be a consequence of proliferative DR (PDR), such as vitreous hemorrhage from neovascularization, proliferating membrane formation, and tractional retinal detachment, or neovascular glaucoma [113]. Development of drugs that target VEGF such as bevacizumab, ranibizumab and aflibercept have changed the treatment management involved with DME and have had a significant role in the management of DR. These anti‐VEGF drugs have been reported to be safe and effective in multiple clinical trials. Despite their efficacy, there are a proportion of patients who have an incomplete response to therapy [73,114].

Xin et al. [52] first confirmed ANGPTL4 as a possible vasoactive cytokine that may contribute to the promotion of vascular permeability and macular edema (ME), in patients with ischemic retinal disease. They demonstrated that hypoxia induces ANGPTL4 in retinal Müller glial cells, furthermore, in the retina of ischemic retinal disease models and in eyes of patients with diabetic eye disease, the expression of ANGPTL4 is considerably high [52]. ANGPTL4 neutralizing antibody could inhibit the aqueous vasoactive effect in patients with PDR, in samples from patients with either low VEGF levels or patients who had received anti-VEGF therapy, suggesting that targetting both ANGPTL4 and VEGF may be essential for more effective management of DME and PDR [73]. Kwon et al. [115] observed that patients with DME secondary to DR had significantly higher aqueous ANGPTL4 levels than the control cataract group and aqueous ANGPTL4 levels related positively with the severity of ME.

Lu et al. [116] observed that ANGPTL4 levels were obviously increased and the ANGPTL4 expression was related to the VEGF expression in the vitreous and serum of patients with PDR. ANGPTL4 levels were also markedly correlated with serum lipids in patients with PDR which suggest that ANGPTL4 may be used as a potential therapeutic target for the treatment of DR [116]. In a follow-up experiment, they demonstrated that ANGPTL4 regulates diabetic retinal inflammation and angiogenesis by, at least partly, activating profilin-1 both in human retinal microvascular endothelial cells (HRMEC) and in diabetic rats. They also showed that the activation of the ANGPTL4 was dependent on the overexpression of its upstream mediating factor, HIF-1α, under high-glucose conditions both *in vivo* and *in vitro.* This finding calls for future studies to identify a pharmacological inhibitor of ANGPTL4, alone or in combination with an inhibitor of the profilin-1 signaling pathway, as a therapeutic target and diagnostic screening biomarker for PDR and other vitreous-retinal inflammatory diseases [117].

It is important to note that there is contradiction between the relationship of the VEGF and ANGPTL4. Sodhi and co-workers [118] reported that aqueous ANGPTL4 levels were increased in patients who had been treated with anti-VEGF and in which VEGF levels were markedly reduced, implicating that ANGPTL4 expression was independent of VEGF level. However, Lu et al. [116] illustrated that the ANGPTL4 expression was directly correlated with the VEGF expression in the vitreous and serum of patients with PDR*.* Later, Lu et al. [117] assessed the role of ANGPTL4 in the changes in VEGF expression levels under high glucose conditions in HRMECs, with the knockdown of ANGPTL4, *VEGF* mRNA and secretion declined indicating the upstream role for ANGPTL4 with respect to VEGF.

### Branch retinal vein occlusion

Branch retinal vein occlusion (BRVO) is a relatively frequent retinal vascular disorder of elderly people, the resulting ME is the most common cause of visual loss [119]. Kim et al. [120] found that patients with ME due to BRVO had markedly higher aqueous ANGPTL4 levels than the control patient group with cataracts. Furthermore, ANGPTL4 levels in patients with BRVO correlated positively with both central subfield macular thickness (CSMT) and total macular volume (TMV) which are indexes of macula edema, and are increased by vascular hyper-permeability [120]. These results suggest that ANGPTL4 may be a possible biomarker for the severity of retinal ischemia in patients with ME due to BRVO.

### Branch retinal artery occlusion

Sodhi co-workers [121] discovered that an ischemic branch retinal artery occlusion (BRAO) demonstrated progression of retinal NV despite scatter laser treatment and addition of anti-VEGF therapy resulted in transient regression of NV. Conversely, levels of ANGPTL4 were markedly increased in this patient compared with the control, which supports future studies examining the effect of ANGPTL4 in the development of retinal NV in patients with ischemic BRAOs [121].

### Age-related macular degeneration

Age-related macular degeneration (AMD) is one of the most prevalent causes of irreversible vision impairment amongst the elderly people in the Western world. The severity and socio-economic effect of AMD combined with its increasing occurrence requires immediate attention [122]. Ioanna et al. [123] found elevated plasma levels of ANGPTL4 in dry AMD patients suggesting new therapeutic target for dry AMD, a finding consistent with the conclusions of the study of Park and co-workers [124] which investigated the relationship between aqueous ANGPTL4 levels and clinical features in neovascular AMD, and found that ANGPTL4 level was related to the lesion area and the frequency of anti-VEGF treatment. Both studies suggested that ANGPTL4 may be a possible diagnostic and therapeutic biomarker in AMD.

### Sickle cell retinopathy

Sickle cell retinopathy (SCR) is the most common ophthalmologic complication of sickle cell disease (SCD), a hemoglobin disease affecting adults and children. According to existing studies, the ocular manifestation of SCD is vaso-occlusive and affects every vascular bed. Conventional treatment for SCR includes chemotherapy, intravenous phlebotomy to reduce total HbS red cells. In serious cases, laser-mediated photocoagulation and surgery are required [125]. Jee et al. [126] observed increasing expression of ANGPTL4 *in autopsied eyes, aqueous and vitreous samples of* proliferative SCR (PSR) patients compared with controls. This suggests that ANGPTL4 contributes to the development of retinal angiogenesis in sickle cell patients and could therefore be a potential avenue for the treatment of PSR [126].

### Pterygium

A pterygium is a non-neoplastic, degenerative, fibrovascular proliferation of conjunctival tissue that extends into the cornea. Surgical excision remains the most effective intervention but recurrences remain common [127]. Meng et al. [128] reported that HIF-1a accumulation in primary rabbit conjunctival epithelial cells promotes ANGPTL4 expression, and inhibition of ANGPTL4 expression is sufficient to inhibit the angiogenic phenotype of these cells. They further demonstrated that ANGPTL4 expression is observed in the conjunctival epithelium of surgically excised pterygia. These observations suggest that pharmacotherapy independently targetting VEGF and ANGPTL4, or targetting HIF-1 to inhibit both, may be a more effective anti-angiogenic treatment for patients with pterygia [128].

### Uveal melanoma

Melanocytes within the uveal tract lead to uveal melanoma (UM), the most prevalent primary intraocular malignancy amongst adults [129]. Hu et al. [130] discovered that vitreous samples from UM patients who had an incomplete response to anti-VEGF treatment had elevated levels of ANGPTL4. Using a tumor array, they demonstrated an increase in ANGPTL4 in almost 80% of UM tumors, with elevating expression of either VEGF or ANGPTL4 in 99% primary UM tumors. They also observed that ANGPTL4 is involved in the promotion of neovascularization in UM *in vitro* and *in vivo* [130]. Ness et al. [131] demonstrated that the multicellular tumor spheroids (MCTS) cultures from UM patients showed metabolic shift traits related to anoikis resistance as demonstrated by an increase in ANGPTL4 which implies that ANGPTL4 might play an important role in orchestrating lipid metabolism in MCTS. These findings sustain the potential role for ANGPTL4 in the promotion of metastasis in UM and provide a basis for future investigations to determine more effective therapies like combining inhibition of both ANGPTL4 and VEGF to simultaneously target tumor-induced angiogenesis and metastasis.

### The role of the ANGPTL4 in vascular leakage

The establishment and maintenance of vascular function depends on appropriate vascular maturation, endothelial junction remodeling, and perivascular cell recruitment. In pathological circumstances such as ischemic retinopathy (retinopathy of prematurity, DR, and AMD), angiogenesis is accompanied by vascular barrier disruption, which leads to plasma leakage and retina edema [132]. Ito et al. [133] found that ANGPTL4 suppressed the proliferation, tubule formation, and migration of endothelial cells. Furthermore, utilizing corneal neovascularization and Miles permeability assays, they found that ANGPTL4 significantly inhibited angiogenesis and vascular leakiness induced by VEGF *in vivo* [133]. Perdiguero et al. [134] first discovered that ANGPTL4 knockout mice presented with substantially increased leakage of retinal capillaries with a reduction in pericytes, perturbation of caveolae, VE-cadherin, and ZO-1. This study showed that hypoxia-induced angiogenesis vascular leakage led to a feedback loop via expression of ANGPTL4 which limits plasma leakage and endothelial cell disorganization. However ANGPTL4 does not participate in pericyte recruitment, although pericytes fail to spread on ANGPTL4-deficient endothelial cells [134]. In further studies, Perdiguero et al. [134] reported that ANGPTL4 neutralized hypoxia-induced vascular permeability through integrin αvβ3 binding. Stimulation of VEGFR2–Src kinase signaling leads to endothelial junction stabilization, demonstrating therapeutic strategies using ANGPTL4 aimed at controlling vascular permeability in ischemic and ocular diseases [12].

## Conclusions and perspectives

Multiple developmental and pathological roles related to eye diseases are attributable to ANGPTL4 including the promotion of angiogenesis, vascular permeability, and inflammation. However, the study of ANGPTL4 functionality is in its infancy, leading to controversy and a lack of comprehensive investigation regarding the precise role(s) of ANGPTL4 in various disease processes. This is even more complicated by the distribution of alternative cellular functions to three cleavage isoforms of ANGPTL4. Further investigation of ANGPTL4 functions in different pathologies is yet to be performed. It is essential to consider multiple cleavage products as well as the combined effects of modifying various diseases processes on overall disease activity. Luo et al. [135] doubt the safety and application value of developing anti-ANGPTL4 therapy because 93% perinatal mortality was found in newborn ANGPTL4 knockout mice, suggesting that ANGPTL4 may be indispensable for normal embryonic development. Bouleti et al. [136] hypothesized that although until now developing therapeutic agents aimed at blocking ANGPTL4 is intensely debated. ANGPTL4 recombinant protein might be an effective therapeutic target in acute ischemic cardiovascular diseases because of its role in protecting vascular integrity [136]. Until now, ELISA, Western blot, and quantitative PCR have been the preferred techniques for ANGPTL4 detection ascribed to its easy-use, accuracy, and high-throughput, but these techniques ignore distinction of the different ANGPTL4 isoforms. Future investigation of ANGPTL4 should be dedicated to the detection and identification of the various ANGPTL4 isoforms in order to reveal the structure–function relationship of this protein. Immunoblot of lysate, secretion medium, and ECM using anti-cANGPTL4 antibody and anti-nANGPTL4 antibody should be applied at the same time.

Despite a lack of comprehensive knowledge, ANGPTL4 must be considered to be an attractive potential therapeutic tool. A blockade of ANGPTL4 might obviously affect disease progression via inhibition of multiple processes and signal pathways. Promisingly, ANGPTL4 is hypothesized to be a new therapeutic target when used as a recombinant protein. Before, HIF-1α was to be considered to be a potential target for treatment of ischemic retinopathies. However, development is thought to be limited by the lack of drugs that specially block the HIF pathway, limiting further analysis of specific effects of HIF inhibition on ischemic retinopathies progression. Neutralizing anti-ANGPTL4 antibodies have been utilized in murine models of disease and, as interest grows in targetting ANGPTL4 therapeutically, humanized neutralizing anti-ANGPTL4 antibodies will be developed in the near future [137]. In our view, new investigations should not only focus on in-depth molecular mechanism but also on the selective development of new compounds and drugs against specific ANGPTL4 isoforms.

## Abbreviations

AMD: age-related macular degeneration
AMPK: 5′-monophosphate (AMP)- activated protein kinase
ANGPTL4: angiopoietin-like protein 4
BRAO: branch retinal artery occlusion
BRVO: branch retinal vein occlusion
CAD: coronary artery disease
DM: diabetes mellitus
DME: diabetic macular edema
DR: diabetic retinopathy
ERK: extacellular signal-regulated kinase
ECM: extracellular matrix
FOXO1: forkhead box O1
FAK: focal adhesion kinase
FLD: fibrinogen-like domain
HDL: high-density lipoprotein
HIF-1α: hypoxia inducible factor-1
HMEC: human dermal microvascular endothelial cell
HRMEC: human retinal microvascular endothelial cell
HUVEC: human umbilical vein endothelial cell
IL: interleukin
LPL: lipoprotein lipase
MCTS: multicellular tumor spheroid
ME: macular edema
NV: neovascular
NF-κB: nuclear factor κB
PDGF: platelet-derived growth factor
PAK: p21-activated kinase
PDR: proliferative DR
PGE2: prostaglandin E2
PPAR: peroxisome proliferator activated receptor
PSR: proliferative sickle cell retinopathy
Rac1: ras-related C3 botulinum toxin substrate
SCD: sickle cell disease
SCR: sickle cell retinopathy
Src: sarcoma gene
SIRT1: sirtuin 1
TGF-β: transforming growth factor-β
TTP: tristetraprolin
UM: uveal melanoma
VCAM-1: vascular cell adhesion molecule-1
VEGF: vascular endothelial growth factor
WAT: white adipose tissue
